# Development of Glypican 3–Targeting Antibody–Drug Conjugates for Hepatocellular Carcinoma Therapy

**DOI:** 10.1158/2767-9764.CRC-26-0139

**Published:** 2026-07-14

**Authors:** Ning Li, Yu Zeng, Xiaolin Xiong, Peng Zhang, Xiaolun Peng, Wenbin Chen, Wen Wei, Xiaozhen Xia, Ruihong Liu, Yao Lu, Mengyuan Geng, Xianqing Lan, Xiuqi Li, Tingting Yuan, Ying Huang, Ying Xiao, Zongchun Mou, Junming Yie

**Affiliations:** 1 Biomedical Innovation and Discovery Center, Salubris (Chengdu) Biotechnology Co., Ltd., Chengdu, China.; 2 Department of Pharmacology and Toxicology, Shenzhen Salubris Pharmaceuticals Co., Ltd., Shenzhen, China.; 3 School of Biomedical Engineering, Harbin Institute of Technology (Shenzhen), Shenzhen, China.; 4 Biomedical Innovation and Discovery Center, Shenzhen Salubris Pharmaceuticals Co., Ltd., Shenzhen, China.; 5 Biologics Pharmaceutical R&D Center, Salubris (Chengdu) Biotechnology Co., Ltd., Chengdu, China.

## Abstract

Hepatocellular carcinoma (HCC), a leading cause of global cancer mortality, is characterized by intratumoral heterogeneity, chemoresistance, and limited treatments. Glypican 3 (GPC3), an oncofetal glycoprotein overexpressed in ∼70% of HCC cases but absent in normal adult tissues, represents an attractive target for antibody–drug conjugates (ADC). To address this unmet need, three GPC3-specific antibodies were developed: a fully human Mab-A with moderate affinity (Kd = 45.7 nmol/L), recognizing a unique epitope distal to GPC3’s membrane region; a humanized Mab-B with high affinity (Kd = 8.84 nmol/L), targeting an epitope similar to those of several known therapeutic antibodies; and a biparatopic BpAb-AB with the highest affinity (Kd = 0.383 nmol/L). Three payloads were used for conjugation: duocarmycin SA (DUBA), pyrrolobenzodiazepine (PBD) dimer, and deruxtecan (Dxd). In cell line–derived xenograft (CDX) models of HCC, DUBA conjugates demonstrated satisfactory efficacy but less potent than other conjugates, whereas PBD dimer conjugates showed potent tumor suppression with narrow therapeutic windows due to dose-limiting toxicity. Mab-B conjugates exhibited comparatively lower efficacy and suboptimal pharmacokinetics. In contrast, Mab-A-Dxd and BpAb-AB-Dxd ADCs produced sustained tumor regression and acceptable safety. Despite lower binding affinity and *in vitro* activities, Mab-A-Dxd outperformed BpAb-AB-Dxd *in vivo*, driven by superior tumor penetration, prolonged exposure, and reduced target-mediated drug disposition (TMDD). Efficacy was further validated in patient-derived xenograft (PDX) models. Collectively, the optimized Mab-A-Dxd ADC, engineered with high drug-to-antibody ratio (DAR∼10) and Fc-silencing mutations, achieves a favorable balance among tumor penetration, minimized TMDD, and effective GPC3-mediated delivery, positioning it as a promising therapeutic candidate for refractory HCC and other GPC3-expressing malignancies.

**Significance:**

We identified Mab-A, a moderate-affinity GPC3 antibody targeting a unique membrane-distal epitope. Combined with a high-DAR (10) Dxd payload and engineered Fc, Mab-A-Dxd shows superior efficacy in CDX/PDX models, balancing internalization, cytotoxicity, tumor penetration, exposure, and reduced TMDD, redefining ADC design principles and supporting therapeutic potential in HCC and beyond.

## Introduction

Hepatocellular carcinoma (HCC) represents a major global health threat owing to its high incidence, mortality, and recurrence rates (1, 2). Key risk factors include chronic hepatitis B and C viral infections, alcohol abuse, and obesity, with cirrhosis serving as the principal predisposing condition (3, 4). Current treatment paradigms remain suboptimal, relying largely on multidisciplinary approaches that often combine multimodal therapies such as surgical resection, ablation, transarterial chemoembolization or transarterial radioembolization, transplantation, and systemic treatments (5, 6). Despite recent advances, long-term clinical outcomes remain disappointing; molecularly targeted agents such as the first-line tyrosine kinase inhibitor sorafenib show limited efficacy, with objective response rates (ORR) of only ∼5%, whereas immunotherapy-based combinations have improved ORR to ∼30%, yet durable responses remain rare and acquired resistance is common (7, 8). This therapeutic landscape underscores the urgent clinical need for innovative therapeutic strategies, with antibody–drug conjugates (ADC) emerging as a promising approach for HCC treatment (9–11).

Glypican-3 (GPC3) is a conserved heparan sulfate proteoglycan with a molecular mass of ∼70 kDa, comprising a core protein, two heparan sulfate (HS) chains, and a glycosylphosphatidylinositol (GPI) anchor that tethers it to the cell membrane (12). The human GPC3 (hGPC3) HS side chains are attached to Ser495 and Ser509 within the C-terminal region, whereas Ser560 serves as the GPI anchor site (13, 14). GPC3 is predominantly expressed during embryonic and fetal development, in which it plays a critical role in regulating organogenesis and tissue patterning (15, 16). In healthy adult tissues, GPC3 expression is absent or at low levels (17, 18). Aberrant GPC3 overexpression is observed in multiple malignancies, notably HCC (70%–85% of cases), as well as non–small cell lung cancer (NSCLC), melanoma, and gastric cancer (12, 19).

*GPC3* is recognized as an oncogenic gene in HCC by coordinating multiple protumorigenic signaling cascades (14, 20). In the Wnt signaling pathway, GPC3 serves as a Wnt coreceptor to enhance Wnt/Frizzled ligand–receptor interactions, facilitating pathway activation, thereby inducing transcriptional upregulation of proliferation-associated genes such as cyclin D1 (13, 21). In the insulin-like growth factor (IGF) pathway, GPC3 amplifies IGF1-mediated oncogenic signaling through enhanced ligand–receptor binding to prevent IGF1R degradation and enhance IGF1-mediated HCC cell growth (17, 22). Clinically, elevated GPC3 expression correlates with aggressive HCC phenotypes, including increased vascular invasion, higher recurrence rates, advanced malignancy grades, and poor patient prognosis (17, 23). Collectively, these characteristics establish GPC3 as a highly selective and clinically actionable target for cancer therapies. Thus, multiple GPC3-targeted therapeutic antibodies have been developed. Codrituzumab (GC33; Chugai Pharmaceutical), the first antibody developed to target GPC3 (24), however, failed to yield significant clinical benefit in patients with HCC in recent trials (25, 26), suggesting that its causal role in oncogenesis may not be as robust as initially proposed.

In addition, owing to the HCC-specific expression profile of GPC3, biopharmaceutical enterprises are actively pursuing GPC3-targeted immunotherapeutic strategies for HCC treatment. Anti-GPC3/CD3 bispecific antibodies, such as ERY974 (Chugai Pharmaceutical; ref. 27) and CM350 (Keymed Biosciences), as well as the anti-GPC3/T-cell receptor nanobody SAR444200 (Sanofi; ref. 28), facilitate the formation of an immune synapse between T cells and tumor cells, thereby promoting T cell–mediated cytotoxicity against GPC3-overexpressing tumors. Chimeric antigen receptor (CAR)–based cell therapies targeting GPC3 represent another attractive approach, with multiple engineered platforms advancing into early clinical trials (29). However, serious or even life-threatening side effects are frequently observed in the use of bispecific T-cell engager (BiTE) or CAR T-cell therapy, most notably cytokine release syndrome (11, 29). Moreover, the unfavorable HCC tumor microenvironment also creates a physical barrier for immune cell infiltration, further limiting the efficacy of these T cell–based therapies (29, 30). Reducing treatment-related toxicities while improving therapeutic efficacy therefore remains a major clinical challenge.

ADCs, which combine the targeted precision of antibodies with the potency of cytotoxic payloads, have achieved remarkable clinical success in solid tumors (31). Accordingly, ADCs are considered a promising therapeutic strategy for HCC (9, 10). In order for this strategy to work, ADCs must undergo efficient target-mediated internalization to enable intracellular release of the active drug payload. It is known that GPC3 can be rapidly internalized upon binding to hedgehog (Hh) proteins through a process mediated by low-density lipoprotein receptor–related protein 1 (LRP1; refs. 9, 32). This intrinsic internalization capability, together with its selective overexpression in HCC, renders GPC3 an attractive target for ADC development. Consistent with this rationale, several anti-GPC3 ADCs have recently advanced into early clinical stage, including ZW251 (Zymeworks), MRG006A (Lepu Biopharma), and BC2027 (BioCity Biopharma). Furthermore, the combination of ADCs and other HCC therapies, such as immune checkpoint inhibitors, hold substantial promise for further improving overall therapeutic outcomes.

This study focuses on the preclinical development of an innovative ADC targeting GPC3. To assess the therapeutic potential of GPC3-directed ADCs for HCC, both monoparatopic and biparatopic antibodies with various affinities were generated to construct different ADC formats. These antibodies were conjugated to different cytotoxic payloads, including duocarmycin SA (DUBA), pyrrolobenzodiazepine (PBD) dimer, and deruxtecan (Dxd). A comprehensive preclinical evaluation was conducted to characterize these ADC candidates. *In vitro* assays were performed to assess target-mediated endocytosis and cytotoxicity in GPC3^+^ hepatoma cell lines, as well as *in vivo* efficacy studies in murine cell line–derived xenograft (CDX) and patient-derived xenograft (PDX) models. During the studies, we observed an intriguing disconnection between *in vitro* cytotoxicity potency and *in vivo* tumor growth inhibition.

## Materials and Methods

### Mice

All animals were purchased from GemPharmatech Co., Ltd. Experimental protocols were approved by the Institutional Animal Care and Use Committee (IACUC) and conducted in strict accordance with the Guidelines for Ethical Review of Welfare and Ethics of Laboratory Animals (GB/T 35892-2018) and GemPharmatech’s standardized operating procedures. Both male and female BALB/c nude mice at 6 to 8 weeks of age were used for experiments. Animals were housed in specific pathogen–free–grade barrier facilities with a 12-hour light/dark cycle, controlled temperature (20°C–26°C), humidity (30%–70%), and high-efficiency particulate air (HEPA)-filtered ventilation. Each cage (size: 32 × 20 × 18 cm) contained autoclaved bedding (corncob), environmental enrichment (nesting material), and *ad libitum* access to sterile food and 0.2 μm–filtered reverse osmosis water to prevent microbial contamination.

### Production of GPC3 monoclonal or biparatopic antibodies

BALB/c mice were immunized with the full-length extracellular region of hGPC3 (amino acid positions between Gln25 and His559). Hybridoma clones producing monoclonal antibodies (mAb) with strong binding affinity to the antigen were screened by enzyme-linked immunosorbent assay (ELISA), and the variable region sequences of the antibodies were characterized using the rapi5′-rapid amplification of cDNA ends (RACE) method using FirstChoice RLM-RACE Kit (Thermo Fisher Scientific, AM1700). Of them, mMab-B antibody was selected for humanization by complementarity-determining region grafting. The humanized antibody was named Mab-B.

To obtain antibodies with different epitopes on GPC3, a C-terminal region peptide was synthesized (Bankpeptide) and conjugated to a carrier protein, keyhole limpet hemocyanin (KLH) or bovine serum albumin (BSA), via a cysteine residue introduced at the N-terminus. Phage-display screening was then performed using a humanized recombinant antibody library (Sanyou Biopharmaceuticals), leading to the identification of a GPC3-specific antibody Mab-A.

Epitope mapping was performed using synthesized peptides. Each peptide contains a sequence of 12 amino acids that spans the C-terminal region of GPC3 between Ser477 and Ser560 and overlaps with adjacent peptides by eight residues (Supplementary Table S1). The binding of Mab-B and Mab-A to these peptides was tested by ELISA, and distinct epitopes were identified.

The biparatopic antibody was designed by fusing the Mab-A single-chain variable fragment (scFv) to the N-terminus of the light chain of Mab-B to minimize steric hindrance, and the heavy chain remained unchanged. All of these three antibodies, namely, humanized antibody Mab-B, fully human antibody Mab-A, and the biparatopic antibody BpAb-AB, were reformatted into human IgG1 (hIgG1) with engineered mutations in the Fc region for Fc silencing (L234A, L235A, and P329G; refs. 33, 34) and site-specific conjugation (S239C).

Binding affinities of Mab-A, Mab-B, BpAb-AB, ZW251-Ab (WO2024082051A1), MRG006A-Ab (WO2025051159A1), GC33 (US20150259417A1), and YP7 (US9409994B2) were characterized using ELISA and surface plasmon resonance (SPR; Biacore 1K). For ELISA, 96-well plates coated with hGPC3 (2 μg/mL) were incubated with serially diluted antibodies (0.001–100 nmol/L), followed by horseradish peroxidase–conjugated anti–human IgG detection and EC_50_ calculation via nonlinear regression. SPR analysis was performed on a Biacore 1K using a Protein A chip with hGPC3. Antibodies were injected to measure association/dissociation rates and equilibrium dissociation constants (Kd) using a 1:1 Langmuir binding model.

### Synthesis of ADCs

Antibodies Mab-A, Mab-B, and BpAb-AB were conjugated to tesirine (MedChemExpress, HY-128952; ref. 35), valine–citrulline–seco-DUBA (Vc-seco-DUBA, MedChemExpress, HY-128957) or Dxd (MedChemExpress, HY-13631E) using the maleimide–thiol reaction. For tesirine and Vc-seco-DUBA, site-specific conjugation was achieved by attaching the payload to an engineered cysteine residue (Cys239) in the antibody heavy chain. In contrast, Dxd conjugates were generated via random conjugation to reduced cysteines to achieve a high drug-to-antibody ratio (DAR). Reducing agents cleave interchain disulfide bonds in antibodies, generating free cysteine residues and exposing reactive thiol groups (-SH), which undergo Michael addition with maleimides at the linker–payload terminus. This enables covalent attachment of Dxd to the antibody through stable thioether bonds (36). In parallel, engineering the S239C mutation in the Fc region allows a theoretical maximum DAR of 10. The DAR value was determined using reversed-phase ultra-high performance liquid chromatography (Vanquish system, RRID: SCR_025713) coupled with high-resolution mass spectrometry (Q Exactive, RRID: SCR_020566). Briefly, the ADC sample was reduced and deglycosylated prior to analysis. The deconvoluted mass spectra were assigned to the light and heavy chains bearing different numbers of linker–payloads, and the final DAR was calculated as the intensity-weighted average of the identified species. ADCs using tesirine or Vc-seco-DUBA payload were designated Mab-A-PBD, Mab-B-PBD, and BpAb-AB-PBD or Mab-A-DUBA, Mab-B-DUBA and BpAb-AB-DUBA, respectively, with observed DAR values ranging from 1.5 to 1.9. ADCs incorporating Dxd were designated Mab-A-Dxd and BpAb-AB-Dxd, with observed DAR values ranging from 8 to 10.

### Cell culture

Three human HCC cell lines HepG2 (RRID: ACC_180), Hep3B (RRID: ACC_93), Huh7 (RRID: JCRB0403), and human Burkitt lymphoma cell line (Raji, RRID: ACC-319) were purchased from American Type Culture Collection. A375 (RRID: CRL_7904) and HEK293 (RRID: CVCL_0045) cell lines were purchased from National Collection of Authenticated Cell Cultures. HEK293-GPC3 (ProBio, RRID: CVCL_0063) is a HEK293 cell line stably expressing hGPC3. All cell lines were authenticated via short tandem repeat profiling, verified by morphology and growth rate, and confirmed to be free of *Mycoplasma* contamination. All cells were cultured in DMEM (Gibco) or RPMI 1640 (Gibco), supplemented with 10% fetal bovine serum (FBS; Gibco), 100 U/mL penicillin (Sigma), and 0.1 mg/mL streptomycin (Sigma-Aldrich), in a cell culture incubator at 37°C/5% CO_2_. All cell lines were passaged fewer than 15 times when used for experiments.

### Cellular binding assay

Flow cytometry was used to analyze the binding of antibodies (Mab-A, Mab-B, and BpAb-AB) or corresponding ADCs (PBD-, DUBA- or Dxd-conjugated) to cell lines with various GPC3 expression levels to determine their cellular binding capability. HepG2 (GPC3^High^), Hep3B (GPC3^Medium^), Huh7 (GPC3^Low^), HEK293 (GPC3^Low^), HEK293-GPC3 (GPC3-overexpressing stable cell line), and Raji (GPC3^−^) cell lines were used in this study. The antibodies or ADCs were diluted with FACS buffer (PBS supplemented with 5% FBS). The cells were suspended in FACS buffer and incubated with the diluted samples for 1.5 hours at 4°C. Cells were washed 3 times with FACS buffer and subsequently incubated with PE-conjugated anti–human IgG (BioLegend, RRID: AB_2565785) at a 1:100 dilution for 30 minutes at room temperature. Following three additional washes with FACS buffer, fluorescence was measured using an Attune NxT Flow Cytometer (Thermo Fisher Scientific, RRID: SCR_019590).

### *In vitro* cell killing and bystander effect assay

Cell lines such as HepG2, Hep3B, Huh7, and Raji were incubated in growth medium with serial dilutions of ADC samples at 37°C in a 5% CO_2_–gassed, humidified incubator. Cell viability was measured with a Cell Counting Kit-8 (CCK-8) kit (Promega) after 5 days. Absorbance values were measured at 450 nm wavelength on a Thermo Fisher Scientific Multiskan GO reader using protocol set up for CCK-8 assays. The 50% inhibitory (IC_50_) values were determined using GraphPad software (GraphPad Prism 8, RRID: SCR_002798).

For the bystander effect assay, GPC3^−^ A375 cells stably transfected with green fluorescent protein (GFP) were used as an indicator for bystander killing. HepG2 (GPC3^+^) and A375 (GPC3^−^) cells were co-incubated and treated with the Mab-A-Dxd, BpAb-AB-Dxd, or isotype control ADC for 5 days. The concentrations of Mab-A-Dxd and BpAb-AB-Dxd were selected to be higher than the HepG2 IC_50_ but lower than the A375 IC_50_. The viability of GFP-labeled A375 (GPC3^−^) cells was quantified by measuring fluorescence intensity.

### Antibody internalization study

The live-cell analyzer Incucyte S3 (Sartorius, RRID: SCR_023147) was used to assess GPC3-mediated antibody internalization. A pH-sensitive fluorescent dye, Incucyte Fabfluor-pH (Sartorius), was used for labeling. This dye exhibits minimal fluorescence under neutral pH (7.4) in standard cultural medium but emits intense red fluorescence upon internalization into acidic endosomes and lysosomes (pH 4.5–5.5). HepG2 cells were treated with Incucyte Fabfluor–labeled anti-GPC3 antibodies (Mab-A, Mab-B, and BpAb-AB) or a hIgG1 isotype control at five concentration points: 60, 20, 6.67, 2.22, and 0.74 nmol/L. Antibody internalization was monitored in real time over 48 hours. Total red object integrated intensity (RCU × μm^2^ per image), defined as the product of relative cytometry units (RCU) and the area of all red fluorescent regions, was used to quantify total antibody internalization. Fluorescence signals were normalized to cell confluence using metrics from the Incucyte live-cell analysis system. Confluence was measured simultaneously with pH-dependent red fluorescence (Excitation/Emission 640/680 nm) using phase-contrast imaging, ensuring that signals were adjusted for variations in cell density over time. This normalization accounts for potential proliferation effects, as Incucyte confluence metrics directly reflect cell-number changes during the assay period (typically ∼24-hour kinetic measurements).

### CDX animal model

Hep3B, Huh7, or HepG2 cells (2 × 10^6^) were suspended in 200 μL of PBS and inoculated subcutaneously into the right flank of 7/8-week-old female/male BALB/c nu/nu nude mice (GemPharmatech Co. Ltd.). Randomization was performed when the average tumor volume (TV) reached approximately 100 to 300 mm^3^ (noted as PG-D0), with six mice per group. The mice were intravenously injected with ADC samples (PBD-, DUBA-, or Dxd-conjugated) at doses described in the figures. TV (mm^3^) was estimated twice per week and calculated using the formula: TV = 0.5 × long diameter × (short diameter)^2^. The treatment-to-control (T/C) value and tumor growth inhibition (TGI) rate were calculated using TV data (see below formula). Relative TV (RTV) = Vt/V0, where V0 represents the individual’s TV on the day of randomization and Vt is its TV after the treatment on the given day. T/C (%): = T_mRTV_/C_mRTV_ × 100%, where T_mRTV_ and C_mRTV_ represent the mean RTV of the treatment and control groups, respectively, on a given day. TGI_TV_ (%) = (1 − T/C) × 100%, with TGI = 100% indicating that the TV in the treatment group is reduced to a size smaller than the baseline TV at the time of initial administration. Mice were euthanized at the termination of the study. Tumors were isolated and weighed, and photographs were taken.

### Pharmacokinetics evaluation in the HCC CDX animal model

For the pharmacokinetics (PK) study (37), Hep3B and Huh7 human hepatocyte cancer cells were inoculated subcutaneously into the right flank of BALB/c nude male mice. Randomization was performed when the average TV reached approximately 300 mm^3^ with six mice per group. The Hep3B CDX model group was used to study the serum concentration change in Mab-A-PBD, Mab-B-PBD, and BpAb-AB-PBD. After a single-dose treatment of 1.5 mg/kg, multiple timepoint analysis of the total antibody concentrations was performed over 45 days for long-term observation. In the Huh7 CDX model, mice were randomized into four groups and single-dosed with equimolar Mab-A-Dxd (1.5 or 6 mg/kg) or BpAb-AB-Dxd (2 or 8 mg/kg) when tumors reached an average volume of ∼300 mm^3^. Serum samples were collected at 5 minutes and 2, 7, 24 (day 1), 48 (day 2), 72 (day 3), 120 (day 5), 168 (day 7), 336 (day 14), and 504 hours (day 21) after dosing to determine the total antibody concentrations of Mab-A-Dxd and BpAb-AB-Dxd.

### Tumor penetration study in the CDX animal model

The distribution of PBD-conjugated ADCs was evaluated in Hep3B xenografts, whereas Dxd-conjugated ADC distribution was assessed in HepG2, Hep3B and Huh7 xenografts. These distribution studies were conducted in parallel with the corresponding efficacy studies. Mice were randomized into 3 or 4 groups (*n* = 2 per group) when tumors reached an average volume of approximately 400 mm^3^. Animals received a single intravenous dose (1.5 mg/kg) of Mab-A-PBD, Mab-B-PBD, BpAb-AB-PBD, or IgG1-PBD or Mab-A-Dxd, BpAb-AB-Dxd (doses indicated in the figure captions), or vehicle control. Tumor tissues were collected at 72 hours (for PBD-conjugated ADCs) or 24 hours (for Dxd-conjugated ADCs) after administration and immediately fixed in 10% neutral buffered formalin for 48 hours prior to histopathologic analysis. Immunohistochemistry (IHC) was performed on tumor sections using a primary anti–human IgG antibody (1:200 dilution; Abcam, ab181236) to assess ADC (total antibody) distribution and tumor penetration. All IHC images were analyzed using the HALO platform, with quantitative assessment restricted to the defined tissue regions of interest. Staining intensity was classified into four categories: 0 (negative), 1^+^ (weak), 2^+^ (medium), and 3^+^ (strong). The percentage of cells at each intensity level was quantified, and an H-score was calculated to integrate both staining intensity and distribution. The H-score was calculated as

$$H‐\text{Score}\;=\;\left(\%\;\text{of}\;\text{cells}\;\text{at}\;0\right)\;\times \;0\;+\;\left(\%\;\text{at}\;1^{+}\right)\;\times \;1\;+\;\left(\%\;\text{at}\;2^{+}\right)\;\times \;2\;+\;\left(\%\;\text{at}\;3^{+}\right)\;\times \;3,\;\;\text{yielding}\;a\;\text{total}\;\text{score}\;\text{ranging}\;\text{from}\;0\;\text{to}\;300.$$

### PDX animal model

Mab-A-Dxd was evaluated in human liver cancer PDX models (LI6610, LI1037, and LI6619) and a human lung cancer PDX model LU1542 (Crown Bioscience). Each mouse was subcutaneously inoculated in the right upper flank with a tumor fragment (2–3 mm in diameter) for tumor development. Mice were randomized into groups (5 mice per group) when the mean tumor size reached approximately 100 to 150 mm^3^.

### PK study of Mab-A-Dxd in non-human primates

To evaluate the *in vivo* PK of Mab-A-Dxd in cynomolgus monkeys as preliminary assessment, Mab-A-Dxd at a single dose of 3 mg/kg was administrated to three monkeys via intravenous injection. Serum samples were collected at 5 minutes and 2, 8, 24, 72, 120, 168, 336, 504, and 672 hours after dosing. Concentrations of total antibody and ADC were determined using ELISA, whereas free payload concentrations were measured using LC/MS-MS.

### Preliminary toxicology evaluation of Mab-A-PBD and Mab-A-Dxd in non-human primates

Dose range–finding studies of Mab-A-PBD and Mab-A-Dxd were performed via intravenous injection in cynomolgus monkeys. As for Mab-A-Dxd, 30 and 60 mg/kg dose groups were evaluated, with one male cynomolgus monkey per group and one additional female in the 60 mg/kg group. Animals were administered once every 3 weeks for a total of three administrations (on day 1, day 22, and day 43), the dosing volume was 5 mL/kg, and the dosing rate was 30 minutes per animal. As for Mab-A-PBD, two doses of 0.6 and 1 mg/kg were evaluated using a study design similar to that described above.

### Statistical analysis

All representative results were reproduced in at least three independent experiments, except for non-human primate (NHP) studies. Data are presented as mean ± standard deviation (SD). All statistical analyses were performed using GraphPad Prism 8 (GraphPad). Analyses followed a structured, hypothesis-driven approach tailored to data distribution and comparison objectives. Normality was first assessed using the Shapiro–Wilk test, and homogeneity of variance was evaluated using the Levene test. For parametric data (Levene test *P* > 0.05), two-way ANOVA was used to assess overall differences among group means, followed by *post hoc* pairwise comparisons using the Tukey honestly significant difference test (all pairs) or Dunnett *t* test (treatment vs. control). For nonparametric data (Levene test *P* ≤ 0.05), the Kruskal–Wallis test was applied to compare group medians, followed by the Conover nonparametric test for *post hoc* analyses (all-pair or many-to-one comparisons). All *post hoc* tests used single-step *P* value adjustment to control type I errors. For pairwise comparisons of PK parameters between two groups, the Student *t* test was used when variance homogeneity was assumed; otherwise, the nonparametric Mann–Whitney U test was applied. This tiered framework ensures robust statistical inference by aligning test selection with data characteristics and study objectives. A *P* value < 0.05 was considered statistically significant. *P* values are indicated in the figures as follows: *, *P* < 0.05; **, *P* < 0.01; ***, *P* < 0.001; ****, *P* < 0.0001; ns, not significant.

## Results

### Anti-GPC3 antibody discovery and characterization

**Figure 1.**
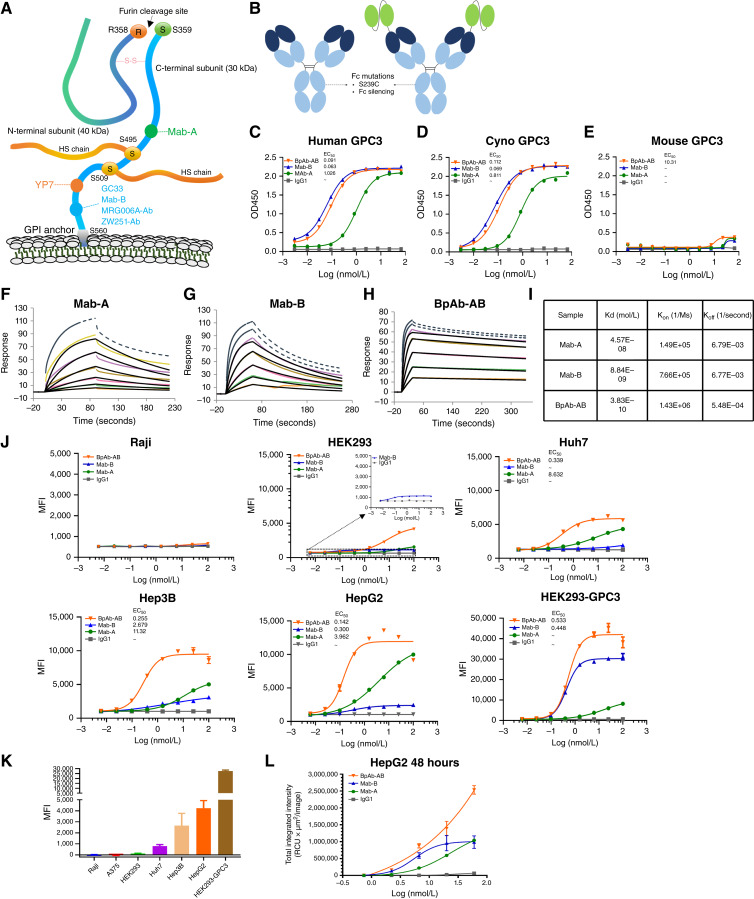
Antibody design and characterization. **A,** Schematic representation of GPC3 epitopes recognized by Mab-A, Mab-B, YP7, GC33, MRG006A-Ab, and ZW251-Ab. **B,** Schematic representation of the mAbs and the biparatopic antibody (IgG–scFv format) incorporating Fc mutations. **C–E,** ELISA-based binding of Mab-A, Mab-B, and BpAb-AB to hGPC3, cynomolgus monkey GPC3 (Cyno GPC3), and mouse GPC3. **F–I,** Biacore kinetic analysis of Mab-A, Mab-B, and BpAb-AB binding to hGPC3. **J,** Flow cytometric analysis of antibody binding to GPC3^+^ hepatoma cell lines (HepG2, Hep3B, and Huh7), the GPC3-overexpressed cell line HEK293-GPC3, the parental HEK293 cell line with very low GPC3 expression, and GPC3^−^ cell line Raji. MFI, mean fluorescence intensity. Data are representative of at least three independent experiments. **K,** Relative GPC3 expression levels across tested cell lines. GPC3 expression was quantified using the GPC3-specific antibody GC33 and an isotype control (IgG1) at 1 nmol/L. Fluorescence signals were normalized to the isotype control to account for nonspecific binding. **L,** Internalization of Mab-A, Mab-B, and BpAb-AB in HepG2 cells, plotted using 48-hour timepoint data.

To generate GPC3-specific antibodies, mice were immunized with the full-length extracellular domain of hGPC3, followed by classical hybridoma screening. Among the candidates, only a single clone (mMab-B) demonstrated desirable binding to GPC3^+^ cells during preliminary screening. This low discovery rate is likely attributable to the high sequence homology (∼94%) between hGPC3 and murine GPC3, which hinders murine antibody generation against hGPC3. The humanized antibody was designated as Mab-B. Epitope mapping revealed that Mab-B recognizes a binding site similar to those of previously reported antibodies, including GC33, MRG006A-Ab, and ZW251-Ab, and is also proximal to the epitope recognized by YP7 (Supplementary Table S1). To identify antibodies with distinct epitope specificity, a phage-displayed human antibody library was screened using a linear peptide derived from the C-terminus of hGPC3. This approach yielded a fully human antibody, Mab-A, which binds a unique epitope not recognized by Mab-B or other known antibodies (Supplementary Table S1; Fig. 1A). Given their distinct epitope specificities, Mab-A and Mab-B were combined to generate a biparatopic antibody (BpAb-AB) with the potential for enhanced binding affinity and therapeutic efficacy in GPC3-targeted ADCs. BpAb-AB was constructed by fusing the scFv of Mab-A to the N-terminus of the Mab-B light chain, resulting in an IgG-scFv bispecific format (Fig. 1B). All three antibodies were engineered with an S239C mutation in the Fc region to enable site-specific conjugation, along with Fc-silencing mutations to minimize off-target cytotoxicity.

Protein-level binding affinities were first assessed using ELISA. Both BpAb-AB and Mab-B demonstrated high binding affinity to hGPC3 and cynomolgus monkey GPC3, with EC_50_ values ranging from 0.06 to 0.11 nmol/L (Fig. 1C and D). In contrast, Mab-A exhibited modest, relatively weaker affinity for hGPC3 (EC_50_ = 1.03 nmol/L) and monkey GPC3 (EC_50_ = 0.81 nmol/L; Fig. 1C and D). None of the antibodies bound to murine GPC3 (Fig. 1E). Consistent with the ELISA results, Biacore kinetic analysis showed that BpAb-AB and Mab-B had high affinity for hGPC3, with Kd values of 3.83 × 10^−10^ and 8.84 × 10^−9^ mol/L, respectively. Mab-A affinity was approximately 10-fold lower than that of Mab-B (Kd = 4.57 × 10^−8^ mol/L), primarily due to a faster dissociation rate (K_off_; Fig. 1F–I).

Flow cytometry was subsequently performed to assess cellular binding across hepatoma cell lines with various GPC3 expression levels. Among the antibodies tested, the biparatopic antibody BpAb-AB exhibited the strongest binding across all expression levels (Fig. 1J and K). Mab-B showed potent and robust binding to HEK293-GPC3 cells (Fig. 1J), which express significantly higher levels of GPC3 than any of the hepatoma cell lines with endogenous GPC3 expression. Interestingly, although Mab-A displayed lower affinity at the protein level, its cellular binding profile differed. Across hepatoma cell lines with various GPC3 expression levels (Huh7, Hep3B, and HepG2 cells), Mab-A showed lower binding potency (higher EC_50_ value) but higher maximal binding compared with Mab-B (Fig. 1J). None of the antibodies bound to GPC3^−^ Raji cells, confirming target specificity. A low level of binding by BpAb-AB was observed in the parental HEK293 cells; however, subsequent analysis detected very low endogenous GPC3 expression, supporting the specificity of this interaction (Fig. 1K).

To investigate antibody internalization, Mab-A, Mab-B, and BpAb-AB were labeled with a pH-sensitive fluorescent dye (Incucyte Human Fabfluor-pH red). Internalization was monitored in HepG2 cells using the Incucyte live-cell analysis system. Dose-dependent increases in internalization were evident for all three antibodies over 48 hours (Fig. 1L). Notably, although BpAb-AB exhibited the strongest internalization, Mab-A reached an internalization rate comparable with Mab-B with no evidence of saturation, unlike Mab-B. Collectively, these results indicated that Mab-A, Mab-B, and BpAb-AB possess distinct cellular binding and internalization characteristics, with Mab-A showing a nonsaturating internalization profile that may be advantageous for ADC delivery.

### Potent antitumor activity of PBD-conjugated ADCs limited by a narrow therapeutic window

**Figure 2.**
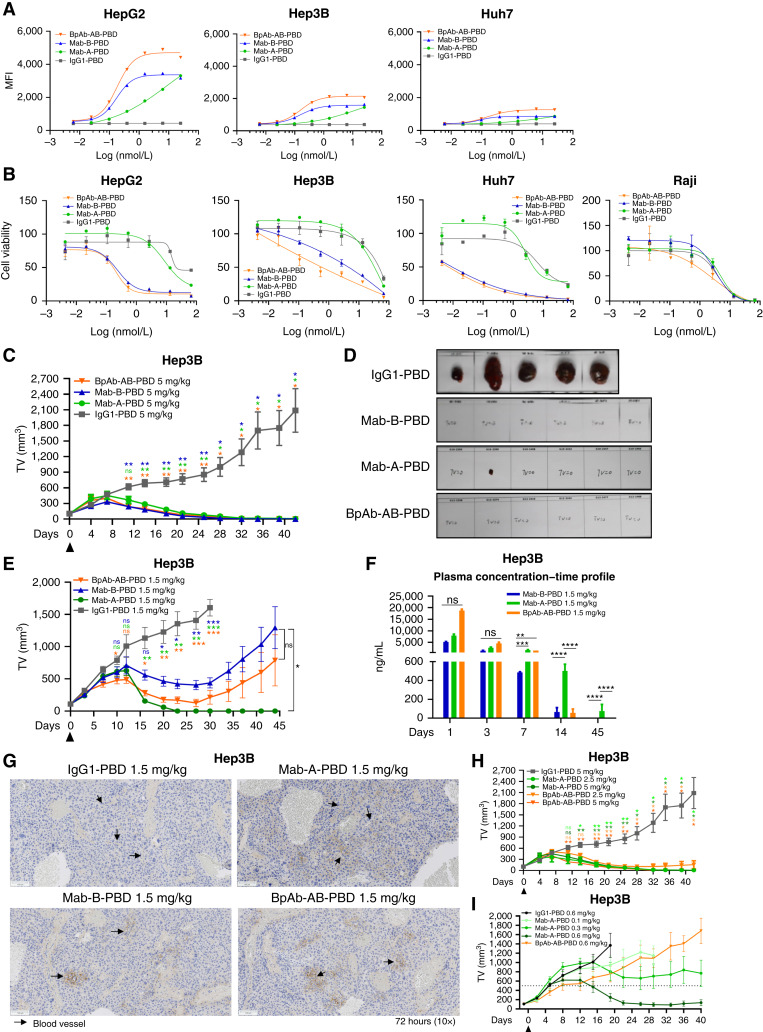
PBD-conjugated ADCs exhibit strong antitumor activity in the Hep3B xenograft model but a narrow therapeutic window. **A,** Cellular binding of Mab-A-PBD, Mab-B-PBD, and BpAb-AB-PBD to GPC3^+^ cell lines (HepG2, Hep3B, and Huh7). MFI, mean fluorescence intensity. **B,***In vitro* growth inhibition of hepatoma cell lines and GPC3^−^ Raji cell line by PBD-conjugated ADCs. Each point represents the mean ± SD (*n* = 3). **C** and **D,***In vivo* antitumor activity of the PBD-conjugated ADCs in the Hep3B CDX model. Tumor growth curves and representative tumor images at the end of treatment. One mouse in the IgG1-PBD group died before the endpoint, precluding tumor imaging on day 48. Each point represents the mean TV and SD (*n* = 6). **E** and **F,** Comparison of antitumor efficacy of Mab-A-PBD, Mab-B-PBD, and BpAb-AB-PBD at a lower dose (1.5 mg/kg) in the Hep3B CDX model, along with corresponding blood concentration profiles (total antibody). Data represent mean ± SD (*n* = 6). **G,** IHC analysis of intratumoral ADC distribution 72 hours after a single dose of 1.5 mg/kg. Magnification, 10×. **H,** Comparative antitumor activity of BpAb-AB-PBD and Mab-A-PBD in the Hep3B CDX model at 2.5 and 5 mg/kg. Data represent mean ± SD (*n* = 6). **I,** Dose–response analysis of Mab-A-PBD *in vivo* efficacy (three dose levels), with 0.6 mg/kg BpAb-AB-PBD included for comparison. Data represent mean ± SD (*n* = 6). *, *P* < 0.05; **, *P* < 0.01; ***, *P* < 0.001; ****, *P* < 0.0001 were considered as significant difference. ns, not significant.

HCC is notorious for its resistance to conventional chemotherapeutic agents. Fu and colleagues (38) reported a large-scale screening of more than 9,000 compounds, in which the DNA-damaging agent PBD dimer exhibited the highest potency against hepatoma cells with subnanomolar IC_50_ values. Inspired by these findings, tesirine, a PBD dimer-based payload, was first selected for ADC development by site-specific conjugation. Flow cytometric analysis confirmed that PBD-conjugated ADCs effectively bound GPC3 on HCC cells, with BpAb-AB-PBD showing the strongest binding, followed by Mab-B-PBD. Mab-A-PBD exhibited weaker binding, and saturation was not reached (Fig. 2A). *In vitro* cytotoxicity assays in HepG2, Hep3B, and Huh7 cells demonstrated that all PBD-conjugated ADCs possessed potent cytotoxic activity, with BpAb-AB-PBD and Mab-B-PBD displaying markedly stronger cytotoxic effects than Mab-A-PBD. In GPC3^−^ Raji cells, no significant differences were observed between anti-GPC3 ADCs and the isotype control ADC, indicating GPC3-specific targeting (Fig. 2B). This observed nonspecific cytotoxicity might be attributed to trace residual free payload (controlled at <0.1%), very low levels of payload release in the medium (39), or nonspecific uptake through pinocytosis (40). Notably, Mab-A-PBD did not show obvious tumor killing activity compared with the control ADC (Fig. 2B), likely due to its weaker cellular binding, with cytotoxicity primarily attributable to the payload rather than antigen-mediated delivery.

The *in vivo* efficacy of PBD-conjugated ADCs was evaluated in a murine Hep3B xenograft model, representing a hepatoma cell line with intermediate GPC3 expression among those tested. One week after a single administration of 5 mg/kg, all PBD-conjugated anti-GPC3 ADCs induced a significant deceleration of tumor growth kinetics compared with the control ADC (*P* < 0.01) and achieved near-complete TGI (approximately 100%; Fig. 2C and D). To further delineate efficacy, a dosing-down study was conducted in the Hep3B xenograft model. At a lower dose of 1.5 mg/kg, Mab-A-PBD achieved sustained TGI (100%), whereas BpAb-AB-PBD and Mab-B-PBD reached TGI rates of 86% and 73%, respectively, by day 27, followed by rapid tumor rebound (Fig. 2E). PK analyses revealed comparable systemic antibody exposure among Mab-A-PBD, Mab-B-PBD, and BpAb-AB-PBD during the first 7 days after administration. However, over a longer period, Mab-B-PBD and BpAb-AB-PBD were cleared more rapidly, with plasma levels becoming undetectable between days 14 and 45, whereas Mab-A-PBD maintained higher circulating levels (Fig. 2F). IHC analysis at the 1.5 mg/kg dose revealed more diffuse intratumoral distribution of Mab-A-PBD, in contrast to the predominantly perivascular location observed for BpAb-AB-PBD and Mab-B-PBD (Fig. 2G). These findings indicate that, compared with the other ADCs, Mab-A-PBD exhibits better tumor penetration and potentially reduced target-mediated drug disposition (TMDD; ref. 41), likely due to its lower binding affinity to GPC3, particularly a faster dissociation rate (K_off_; Fig. 1I).

Dose-escalation studies using Mab-A-PBD and BpAb-AB-PBD at 2.5 and 5 mg/kg in the Hep3B xenograft model showed comparable tumor suppression (Fig. 2H). However, *in vitro* cell toxicity study indicated that subnanomolar PBD dimer is already toxic to cells (Fig. 2B). Therefore, lower doses of Mab-A-PBD were further evaluated *in vivo*. These studies identified 0.6 mg/kg as the minimum dose for complete tumor suppression, whereas lower doses resulted in reduced efficacy. In contrast, BpAb-AB-PBD exhibited minimal efficacy at 0.6 mg/kg (Fig. 2I). Subsequently, a preliminary toxicology study in cynomolgus monkeys was performed to evaluate the safety profile of Mab-A-PBD and established a maximum tolerated dose (MTD) of only 0.6 mg/kg and a lethal dose of 1 mg/kg (Supplementary Table S2). Taken together, the narrow therapeutic index, based on this exploratory cross-species interpretation, and dose-limiting toxicity of Mab-A-PBD render it an unsuitable therapeutic candidate for HCC.

Among PBD-conjugated antibodies with varying affinities, Mab-B-PBD exhibited the weakest antitumor activity (Fig. 2E) and poor PK property (Fig. 2F). In addition, BpAb-AB can provide higher maximal binding and higher affinity than Mab-B if a high-affinity antibody is needed. Consequently, Mab-B was excluded from further development.

### DUBA-conjugated ADCs exhibited strong antitumor activity at high concentrations

**Figure 3.**
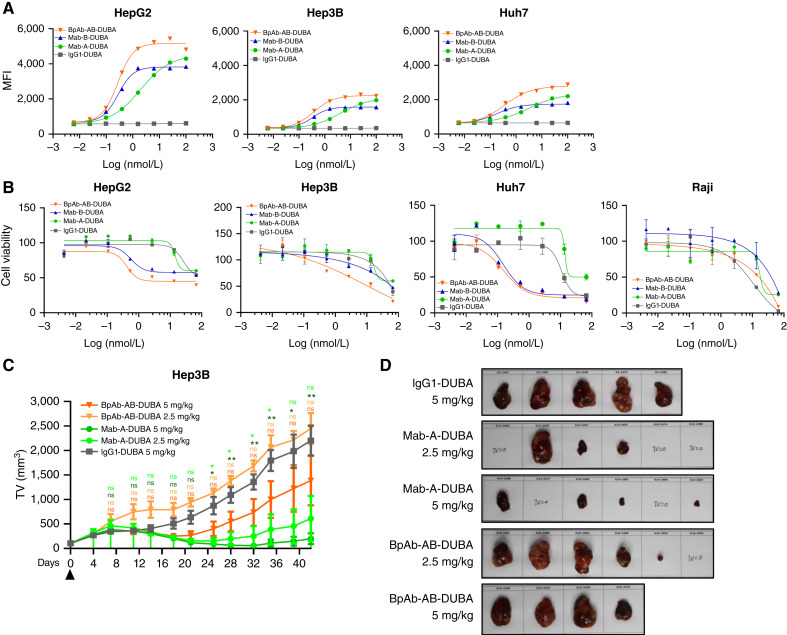
DUBA-conjugated ADCs exhibit antitumor activity at high doses. **A,** Cellular binding of Mab-A-DUBA, Mab-B-DUBA, and BpAb-AB-DUBA to GPC3^+^ cell lines HepG2, Hep3B, and Huh7. MFI, mean fluorescence intensity. **B,***In vitro* growth inhibition of hepatoma cell lines and GPC3^−^ cell line Raji by DUBA-conjugated ADCs. Each point represents the mean ± SD (*n* = 3). **C** and **D,***In vivo* antitumor efficacy of DUBA-conjugated ADCs in the Hep3B CDX model. Tumor growth curves and representative tumor images at the end of treatment. One mouse in the IgG1-DUBA group and two mice in the BpAb-AB-DUBA (5 mg/kg) group died before the endpoint, precluding tumor imaging on day 48. Each point represents the mean TV and SD (*n* = 6). *, *P* < 0.05; **, *P* < 0.01 were considered as significant difference. ns, not significant.

In this study, another DNA-damaging agent, Vc-seco-DUBA (a duocarmycin analogue) reported by Fu and colleagues (38), was selected as an alternative payload. Site-specific conjugation was performed to generate the ADCs. Flow cytometric analysis indicated that DUBA-conjugated ADCs effectively bound to GPC3 on HCC cells, with BpAb-AB-DUBA exhibiting the strongest binding. Mab-B-DUBA showed higher binding potency (lower EC_50_ values) compared with Mab-A-DUBA, whereas Mab-A-DUBA exhibited higher maximal binding capacity (Fig. 3A). *In vitro* cytotoxicity assays in HepG2, Hep3B, and Huh7 cell lines indicated that DUBA-conjugated ADCs exhibited relatively weak cytotoxic activity. In GPC3^−^ Raji cells, similar to PBD-based ADCs, no significant differences were observed between anti-GPC3 ADCs and the isotype control ADC, confirming GPC3-specific targeting (Fig. 3B). Because Mab-B has been excluded, subsequent analyses focused on comparing the *in vivo* efficacy of BpAb-AB-DUBA and Mab-A-DUBA. The *in vivo* efficacy of DUBA-conjugated ADCs was evaluated in a Hep3B xenograft model. The high-affinity biparatopic antibody BpAb-AB conjugated with DUBA did not demonstrate superior antitumor activity; at a high dose of 5 mg/kg, BpAb-AB-DUBA showed weaker efficacy than Mab-A-DUBA at 2.5 mg/kg. BpAb-AB-DUBA also exhibited pronounced tumor regrowth following initial TV regression. Mab-A-DUBA produced dose-dependent TGI but remained suboptimal at 5 mg/kg and did not achieve complete tumor regression (Fig. 3C and D).

Overall, DUBA-conjugated ADCs showed measurable tumor suppression at higher dose levels, with Mab-A-DUBA demonstrating significantly greater antitumor activity than BpAb-AB-DUBA. However, at an equivalent dose of 5 mg/kg, DUBA-based ADCs were generally less effective than their PBD dimer–conjugated counterparts (Figs. 2C, D, 3C, and D). Consequently, DUBA-based ADCs were not pursued for further development.

### Mab-A-Dxd shows effective and sustained tumor suppression activity in GPC3^+^ xenograft models

**Figure 4.**
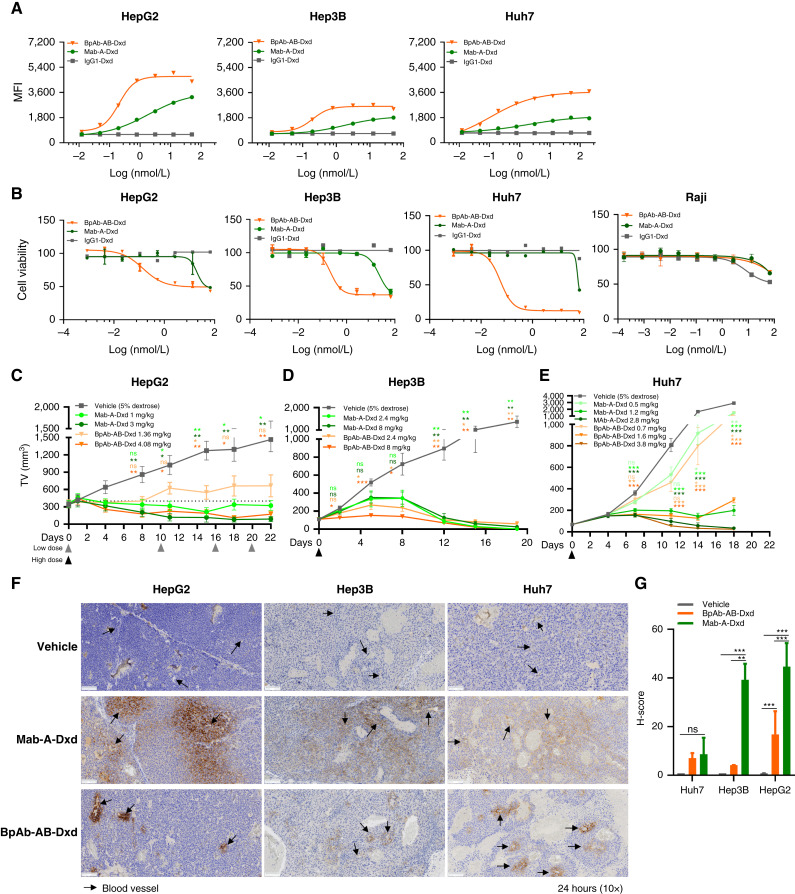
Antitumor activity of Mab-A-Dxd and BpAb-AB-Dxd. **A,** Cellular binding of Mab-A-Dxd and BpAb-AB-Dxd to GPC3^+^ cell lines HepG2, Hep3B, and Huh7. MFI, mean fluorescence intensity. **B,***In vitro* growth inhibition of hepatoma cell lines by Mab-A-Dxd and BpAb-AB-Dxd. **C–E,***In vivo* antitumor efficacy of Mab-A-Dxd and BpAb-AB-Dxd in CDX models with various GPC3 expression levels. In the HepG2 CDX model, low-dose groups received repeated administrations of Mab-A-Dxd (1 mg/kg) and BpAb-AB-Dxd (1.36 mg/kg) on days 10, 16, and 20. Data represent mean ± SD (*n* = 6). **F** and **G,** IHC analysis of intratumoral distribution of Mab-A-Dxd in HepG2, Hep3B, and Huh7 xenografts (1, 2.4, and 1.2 mg/kg, respectively) and BpAb-AB-Dxd (1.36, 2.4, and 1.6 mg/kg, respectively) 24 hours after first dose. Magnification, 10×. All IHC images were quantitatively evaluated and scored using the HALO digital pathology platform. Scoring was performed on whole-slide full-field scanned images, with two independent full-field slides analyzed per group. *, *P* < 0.05; **, *P* < 0.01; ***, *P* < 0.001 were considered as significant difference. ns, not significant.

Given the insufficient efficacy of DUBA-conjugated ADCs and the narrow therapeutic window of Mab-A-PBD, an alternative payload was explored. A clinically validated linker–payload, Dxd (from DS-8201), was then selected as the cytotoxic warhead to produce Mab-A-Dxd and BpAb-AB-Dxd. Dxd, a DNA topoisomerase I inhibitor with nanomolar potency (IC_50_: 0.1–1 nmol/L), demonstrates strong antitumor activity with reduced nonspecific cytotoxicity. Its hydrophilic linker–payload architecture and relatively rapid systemic clearance for the free payload contribute to a markedly improved therapeutic index (42, 43), enabling the implementation of a high DAR. Using the previously described antibody design, including the Fc S239C mutation, random conjugation was performed to achieve a theoretical DAR of 10. Flow cytometric study confirmed that both ADCs effectively bound to HepG2, Hep3B, and Huh7 cells (Fig. 4A). They also demonstrated substantial cytotoxic effects against GPC3-expressing hepatoma cell lines, with BpAb-AB-Dxd showing significantly stronger potency than Mab-A-Dxd (Fig. 4B). In addition, both conjugated ADCs exhibited bystander killing effects (Supplementary Fig. S1A and S1B).

The *in vivo* efficacy of Dxd-conjugated ADCs was evaluated in HCC xenograft models using three hepatoma cell lines with various GPC3 expression levels. Molar equivalent doses of Mab-A-Dxd and BpAb-AB-Dxd were compared to directly assess differences in antitumor activity. Both ADCs demonstrated comparable tumor suppression in a dose-dependent manner, with high doses achieving near-complete inhibition of tumor growth (Fig. 4C–E). In the HepG2 xenograft model, Mab-A-Dxd demonstrated slightly greater tumor growth inhibition. However, neither Mab-A-Dxd nor BpAb-AB-Dxd achieved complete TGI (100%) at lower repeated doses (1 and 1.36 mg/kg, respectively), suggesting that very low doses are insufficient to fully suppress tumor growth to baseline levels (Fig. 4C).

In the Hep3B xenograft model, BpAb-AB-Dxd showed more rapid tumor regression during the first 12 days, but tumor regrowth occurred after 3 weeks at the lower dose of 2.4 mg/kg (Fig. 4D; Supplementary Fig. S2A). IHC analysis showed that Mab-A-Dxd exhibited better tumor penetration, whereas BpAb-AB-Dxd showed a predominantly perivascular accumulation pattern (Fig. 4F and G). These patterns are consistent with those observed for the previously tested PBD-based ADCs. PK analysis in Huh7 tumor-bearing nude mice revealed that Mab-A-Dxd had a significantly longer half-life (T_1_/_2_ = 91.9 ± 15 hours at 1.5 mg/kg and 125 ± 2.2 hours at 6 mg/kg) compared with BpAb-AB-Dxd (T_1_/_2_ = 69.3 ± 12 hours at 2 mg/kg and 87.3 ± 7 hours at 8 mg/kg; Supplementary Fig. S2B; Supplementary Table S3). This difference became more pronounced over time, with BpAb-AB-Dxd showing reduced exposure, particularly at lower doses (Supplementary Fig. S2B), implying a stronger TMDD effect. Consistent with this, in the Huh7 xenograft model, BpAb-AB-Dxd exhibited tumor regrowth after 14 days at a lower dose of 2.4 mg/kg (Supplementary Fig. S2C). Although Mab-A-Dxd has lower affinity for GPC3 and weaker *in vitro* cytotoxicity than BpAb-AB-Dxd, it demonstrated superior tumor penetration, more favorable PK, and robust *in vivo* antitumor activity. As a result, Mab-A-Dxd achieved better efficacy than BpAb-AB-Dxd at low doses, supporting its selection as a more promising candidate for clinical development in HCC therapy.

### Mab-A-Dxd shows high efficacy in GPC3^+^ PDX models

**Figure 5.**
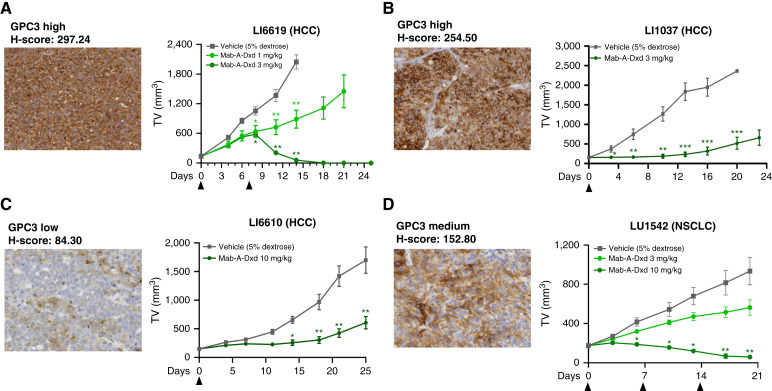
Mab-A-Dxd exhibits strong antitumor activity in GPC3^+^ PDX models. **A–C,** Antitumor efficacy of Mab-A-Dxd in HCC PDX models with various GPC3 expression levels, including LI6619 and LI1037 (high GPC3 H-score) and LI6610 (low GPC3 H-score). Dose levels were selected based on GPC3 expression in each model. **D,** Antitumor activity of Mab-A-Dxd in the NSCLC PDX model LU1542, which exhibits intermediate GPC3 expression (middle GPC3 H-score). Data represent mean ± SD (*n* = 5). *, *P* < 0.05; **, *P* < 0.01; ***, *P* < 0.001 were considered as significant difference.

To further evaluate the *in vivo* antitumor activity of Mab-A-Dxd and its potential as an ADC for HCC, PDX models were employed, as they are considered more clinically relevant because of their use of tumor cells directly derived from patients. In HCC PDX models with high GPC3 expression (H-score, 200–300), including LI6619 and LI1037, Mab-A-Dxd exhibited robust antitumor activity (Fig. 5A and B; Supplementary Fig. S3A). In a low GPC3–expressing HCC PDX model (LI6610; H-score, 1–100), Mab-A-Dxd produced minimal tumor inhibitory effects at 3.1 mg/kg (Supplementary Fig. S3C). However, increasing the dose to 10 mg/kg resulted in significant antitumor activity (Fig. 5C), and this dose was well tolerated in preclinical evaluations (Table 1). GPC3 expression has also been reported in NSCLC, with positivity rates of 66.3% in lung squamous cell carcinoma and 3.3% in lung adenocarcinoma (44). Accordingly, the antitumor activity of Mab-A-Dxd was also assessed in a GPC3^+^ NSCLC PDX model with moderate GPC3 expression (H-score, 100–200). Given the variable and generally lower GPC3 expression in NSCLC (45), two dose levels (3 and 10 mg/kg) were tested. Mab-A-Dxd effectively suppressed tumor growth at 3 mg/kg, whereas 10 mg/kg achieved near-complete tumor inhibition (TGI of approximately 100%; Fig. 5D; Supplementary Fig. S3B), supporting further investigation in other GPC3-expressing malignancies. During those studies, IgG1 isotype control, instead of vehicle control, was added in a few studies but not all to assess target-independent effects. IgG1-Dxd showed only minimal activity compared with placebo, which was negligible relative to the overall efficacy of Mab-A-Dxd (Supplementary Fig. S3A and S3B).

**Table 1.** Summary of Mab-A-Dxd preliminary toxicology studies.

ID	Dose	MTD	TK	Safety window	Toxicity profile
			Total Ab T_1/2_@MTD		
Mab-A-Dxd	30 mg/kg and 60 mg/kg	>60 mg/kg	6∼8 days	>20×	No extra toxicity beyond known Dxd-related effects

### Mab-A-Dxd displays reasonable PK and safety profile in NHPs

**Figure 6.**
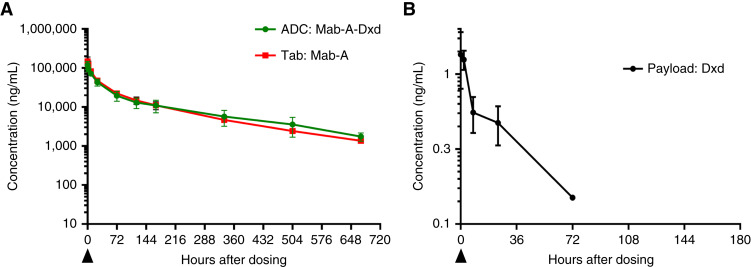
*In vivo* PK of Mab-A-Dxd. **A** and **B,** Plasma PK profiles of the intact ADC (Mab-A-Dxd), total antibody (Tab: Mab-A), and free Dxd following a single intravenous injection of Mab-A-Dxd in cynomolgus monkeys. Data represent mean ± SD (*n* = 3).

Preliminary PK profiling in cynomolgus monkeys at a dose of 3 mg/kg revealed nearly superimposable plasma concentration–time profiles for total antibody and intact ADC, with T_1/2_ = 179 ± 28 hours and T_1/2_ = 192 ± 19 hours, respectively. In parallel, minimal exposure to free Dxd was observed (C_max_ = 1.54 ng/mL; T_1/2_ = 20.5 ± 12 hours; Fig. 6A and B; Table 2). The low free Dxd exposure was likely attributable to trace impurities, as it was rapidly cleared within 3 days. Preliminary toxicology studies in cynomolgus monkeys demonstrated that Mab-A-Dxd was well tolerated up to an MTD of 60 mg/kg, with no additional toxicity beyond known Dxd-related effects, indicating a markedly wide safety window, based on exploratory cross-species interpretation (Table 1; Supplementary Table S4).

**Table 2.** Serum PK parameters of ADC, total antibody, and free payload following administration of Mab-A-Dxd in cynomolgus monkeys.

Analyte	ADC: Mab-A-Dxd	Tab: Mab-A	Payload: Dxd
T_max_ (hours)	0.08 ± 0	0.08 ± 0	0.72 ± 1.1
C_max_ (μg/mL)	148 ± 43	120 ± 28	1.54 ± 0.28
AUC_last_ (h·μg/mL)	7,340 ± 910	7,120 ± 2,000	25.3 ± 12
AUC_INF_ (h·μg/mL)	7,690 ± 870	7,610 ± 2,100	29.8 ± 14
T_1/2_ (hours)	179 ± 28	192 ± 19	20.5 ± 12
T_1/2_ (days)	7.5 ± 1.2	8 ± 0.8	0.9 ± 0.5
Cl (mL/minute/kg)	0.01 ± 0	0.01 ± 0	—
V_ss_ (L/kg)	0.07 ± 0.01	0.08 ± 0.03	—

Abbreviations: AUC_last_, Area under the plasma concentration-time curve from time zero to the last measurable concentration; AUC_INF_, Area under the plasma concentration-time curve from time zero extrapolated to infinity; Cl, clearance; C_max_, maximum concentration; Tab, Total antibody; T_max_, time to maximum concentration; V_ss_, volume of distribution at steady state.

Collectively, these data underscore the favorable PK properties and safety profile of Mab-A-Dxd, supporting its strong potential as an ADC therapeutic for the treatment of HCC and other GPC3-expressing tumors.

## Discussion

GPC3 exhibits high tissue specificity, with more than 70% of HCC and several other tumors showing elevated expression, while remaining minimally expressed in normal human adult tissues, making it an attractive therapeutic target for liver cancer and potentially other malignancies (9, 19, 46). In this study, we identified three antibodies with various binding affinities, namely, a moderate-affinity fully human antibody (Mab-A), a high-affinity humanized antibody (Mab-B), and a biparatopic antibody (BpAb-AB) with the highest affinity. Simultaneously, we evaluated several widely used payloads to address drug resistance in HCC, including PBD dimer, DUBA, and Dxd. The corresponding ADCs were produced and systematically compared across multiple *in vitro* and *in vivo* studies. Unexpectedly, Mab-A-Dxd, the ADC with moderate affinity for GPC3, emerged as the leading candidate owning to its optimal balance of antitumor efficacy and safety.

Although the relationship between antibody affinity and internalization rate is complex and context dependent (47–49), a growing body of evidence suggests that higher affinity often correlates with faster receptor-mediated endocytosis, at least for certain target-antigen systems (47, 48, 50–52), which can significantly affect tumor penetration and therapeutic outcomes (47, 50, 51). High-affinity antibodies targeting GPC3 are associated with enhanced target-mediated internalization, resulting in stronger *in vitro* tumoricidal activity of the corresponding ADCs. However, *in vivo* studies using the Hep3B CDX model revealed that high-affinity ADCs, such as BpAb-AB conjugates, exhibited inferior long-term antitumor efficacy, particularly at low-to-medium dose levels. Even though high-affinity binding promotes rapid internalization and potent cytotoxicity, it can also limit tumor penetration, a phenomenon known as the “binding-site barrier” (47, 50, 53), and is often associated with increased target-mediated clearance, resulting in reduced systemic exposure. In contrast, moderate-affinity ADCs, such as Mab-A conjugates, exhibit relatively slower internalization and lower intrinsic *in vitro* cytotoxicity but achieve improved tumor penetration and prolonged plasma exposure. These properties ultimately translate into more durable antitumor efficacy *in vivo*. The potential impact of antidrug antibodies is considered minimal in this study, as the nude mice used are immunodeficient, lacking functional T cells and exhibiting impaired B-cell responses. Although some differences in binding profiles were observed between unconjugated antibodies and ADCs (Figs. 1J vs. 2, 3, and 4A), the overall trends, lower binding potency of Mab-A compared with Mab-B or BpAb-AB and higher maximal binding of Mab-A compared with Mab-B, remain consistent. This suggests that the inferior long-term efficacy of Mab-B– and BpAb-AB–based ADCs is not primarily due to conjugation-induced changes in binding. Collectively, these findings underscore the importance of selecting antibodies with moderate affinity, a concept that is increasingly recognized and appreciated (54), to balance cell internalization and cytotoxic potency with tumor penetration, systemic exposure, and reduced TMDD.

In addition, even though antibody affinity is a critical determinant of target engagement, it is not the sole factor governing cellular binding. In cell-based binding assays, Mab-B, despite exhibiting much higher affinity for human GPC3 at the protein level than Mab-A, showed a lower maximal binding capacity across all three hepatoma cell lines tested, which span a range of GPC3 expression levels (Fig. 1J and K). Notably, the lower the GPC3 expression, the greater the relative advantage of Mab-A over Mab-B in terms of maximal binding. In contrast, this advantage was not observed in HEK293-GPC3 cells, in which GPC3 is highly overexpressed. A plausible explanation for these observations is the difference in epitope location. Mab-B binds to the A542–H553 region of GPC3, which is proximal to the cell membrane and overlaps with the epitopes recognized by GC33, ZW251-Ab, and MRG006A-Ab, and lies in close proximity to the epitope recognized by YP7. In contrast, Mab-A recognizes the D485–G496 region, which is ∼50 aa more distal from the membrane, potentially allowing greater epitope accessibility and higher binding saturation (Table 3). The biparatopic antibody BpAb-AB, spanning two epitopes, exhibited both the highest binding potency and the greatest maximal binding among the three, although Mab-A may achieve comparable maximal binding at higher concentrations. This enhanced binding saturation may translate into superior TGI and provides a partial explanation for the superior growth inhibition observed with Mab-A– and BpAb-AB–based ADCs compared with Mab-B–based ADCs in the evaluated CDX models.

**Table 3.** Summary of molecular characteristics.

Molecule	Mab-A-Dxd	Mab-B-PBD	BpAb-AB-Dxd	ZW251 (M3-H18L6)	MRG006A (Hu 52H5D3B8)	YP7-PC/DC	GC33
Ab binding epitope	D485–G496 (DKNLDEEGFESG)	A542–H553 (ATPKDNEISTFH)	D485–G496 (DKNLDEEGFESG) + A542–H553 (ATPKDNEISTFH)	A542–H553 (ATPKDNEISTFH)	A542–H553 (ATPKDNEISTFH)	F522–D533 (FLAELAYDLDVD)	A542–H553 (ATPKDNEISTFH)
Ab binding affinity (Kd, by SPR)	4.57 E−08 mol/L	8.84 E−09 mol/L	3.83 E−10 mol/L	9.61 E−09 mol/L	3 E−09 mol/L	6.24 E−10 mol/L	4.69 E−10 mol/L
Payload class	TOPi	PBD dimer	TOPi	TOPi	TOPi	PBD dimer/DUBA	—
Conjugation sites	Interchain disulfides and engineered Cys	Engineered Cys	Interchain disulfides and engineered Cys	Interchain disulfides	Interchain disulfides	Interchain disulfides	—
Theoretical DAR value	10	2	10	4	8	2	—
Fc silence	Yes	Yes	Yes	No	No	No	No

Abbreviation: TOPi, topoisomerase inhibitor.

Payload selection is critical for achieving potent and durable tumoricidal efficacy in HCC (38, 55). DNA-damaging agents such as PBD dimers and duocarmycins, which act as alkylating agents or cross-linkers targeting the DNA minor groove, exhibit exceptional cytotoxicity against HCC cells with picomolar IC_50_ values. However, their clinical utility is limited by significant nonspecific toxicity. For example, repeated dosing of Mab-A-PBD in NHPs resulted in severe off-target toxicity even at low doses (0.6–1 mg/kg), leading to functional impairment of normal tissues and an unacceptable therapeutic index, based on exploratory cross-species interpretation (Supplementary Table S2). In contrast, Dxd, a DNA topoisomerase I inhibitor with nanomolar potency (IC_50_: 0.1–1 nmol/L), demonstrates strong antitumor activity with reduced nonspecific cytotoxicity. Furthermore, its hydrophilic linker–payload architecture and relatively rapid systemic clearance for the free payload contribute to a markedly improved therapeutic index (42, 43), enabling the implementation of a high DAR, specifically DAR = 10 in this study, without precipitating DLT. This represents a key advantage over PBD-based ADCs, which typically require DAR ≤ 4 to mitigate off-target toxicity, yet still failed to achieve an acceptable safety profile in our studies. Consistent with these properties, Dxd-based ADCs demonstrated superior *in vivo* efficacy across multiple CDX and PDX models. Mab-A exhibits a “hit-and-run” binding kinetic profile characterized by a fast association rate (K_on_) and rapid dissociation (K_off_), limiting prolonged cell-surface retention. Although this reduces internalization efficiency, it also decreases TMDD and facilitates deeper tumor penetration, an important advantage in solid tumors such as HCC, in which surface GPC3 expression is high. The combination of Mab-A’s distinctive binding properties with the high-DAR Dxd payload provides several synergistic advantages: (i) The relatively lower intrinsic cytotoxicity of Dxd and low internalization rate of Mab-A are offset by a high DAR (10), enabling effective tumor cell killing; (ii) the moderate affinity of Mab-A enhances solid tumor penetration, promoting greater intratumoral drug distribution; (iii) increased target accessibility due to Mab-A’s membrane-distal epitope, together with high local drug concentration, compensates for reduced internalization and supports effective killing of tumor cells within deeper tumor regions; (iv) the bystander effect of Dxd further amplifies antitumor activity, particularly at higher DAR; and (v) reduced TMDD prolongs systemic exposure, leading to more sustained tumor suppression *in vivo*. Notably, Mab-A-Dxd exhibited excellent tolerability in preliminary toxicology studies in NHPs, achieving a >20-fold safety margin over the efficacious dose.

Off-target toxicities associated with ADCs arise from multiple mechanisms, including linker instability, nonspecific endocytosis, and target-independent uptake mediated by Fcγ receptors (FcγR), the neonatal Fc receptor, and C-type lectin receptors (56, 57). Among these, FcγRs have received particular attention, with accumulating evidence implicating FcγR-mediated interactions as important determinants of ADC safety profiles (56, 58, 59). For instance, thrombocytopenia, a major off-target toxicity of trastuzumab emtansine (T-DM1), had been shown to be mediated, at least in part, by FcγRIIa-dependent internalization into megakaryocytes, thereby impairing their differentiation (60, 61). Interstitial lung disease (ILD)/pneumonitis is a well-recognized toxicity associated with trastuzumab Dxd (T-Dxd), with a reported incidence of approximately 11% to 15%, compared with <2% for T-DM1 (62). Preclinical studies in cynomolgus monkeys have suggested that T-Dxd–related ILD involves target-independent uptake into alveolar macrophages (63). More recent work using an immunocompetent murine model indicates that circulating T-Dxd can be directly engulfed by alveolar macrophages via Fc–FcγR engagement (64, 65). In addition, FcγRs contribute to the update of ADC aggregates or immune complexes into nontarget cells (58, 66). Although Fc-mediated effector functions may contribute to ADC activity, the primary antitumor effect is driven by intracellular delivery of the cytotoxic payload to tumor cells. To reduce off-target uptake and associated toxicity, Fc-silencing mutations were incorporated into the Fc of our ADCs. Although Dxd-based ADCs have demonstrated remarkable clinical efficacy across multiple cancers, systemic toxicities such as ILD and neutropenia remain significant concerns (67). The mechanisms underlying pulmonary toxicities, including ILD, are not fully understood but may involve several factors: (i) low-level target expression in normal tissues; (ii) nonspecific uptake by lung-resident cells such as alveolar macrophages; (iii) systemic release of payload due to premature linker cleavage; and (iv) immune-mediated inflammatory responses (57, 58). Importantly, the Fc silencing alone is unlikely to eliminate all toxicologic risks and remains underexplored (68). Nonetheless, it represents a rational strategy to mitigate unwanted ADC toxicity. Consistent with this approach, several next-generation Fc-silent ADCs are currently under clinical or preclinical investigation, including sofetabart mipitecan (LY4170156), LY4052031, LY4101174, TUB-030, TUB-040, RB-164 (SYS6005), ZW220, and ZW327 (based on publicly available information). In our preliminary toxicology studies of Mab-A-Dxd and Mab-A-DUBA, no obvious ILD was observed in a total of five cynomolgus monkeys (Supplementary Tables S2 and S3). However, these findings are preliminary, and no definitive conclusions can be drawn because of the limited sample size and short observation period. Comprehensive preclinical toxicologic studies and clinical evaluation will be required to rigorously assess the safety of this Fc-silencing strategy.

In conclusion, we identified a unique antibody, Mab-A, that recognizes a GPC3 extracellular epitope located more distal to the cell membrane than those targeted by previously reported GPC3 antibodies, enabling higher maximal binding on GPC3-expressing cells. Mab-A–based ADCs exhibit a balanced affinity that facilitates effective solid tumor penetration and reduced TMDD while maintaining sufficient GPC3-mediated internalization for efficient payload delivery. When conjugated to the high-therapeutic index payload Dxd at a high DAR value (∼10), Mab-A-Dxd demonstrated robust TGI across all evaluated CDX and PDX models. Collectively, these findings support Mab-A-Dxd as a promising therapeutic candidate for the treatment of liver cancer, particularly refractory HCC, and potentially other GPC3-expressing malignancies, warranting further investigation.

## Supplementary Material

Supplementary Figure S1 Bystander killing effects of Dxd-based ADCs.

Supplementary Figure S2 Mab-A-Dxd exhibits superior pharmacokinetics and tumor suppression effect in the CDX model.

Supplementary Figure S3 Mab-A-Dxd displays antitumor activity in GPC3-positive HCC and NSCLC PDX models.

Supplementary Table S1 Epitope mapping of the anti-GPC3 antibodies.

Supplementary Table S2 Mab-A-PBD preliminary toxicology study in cynomolgus monkey.

Supplementary Table S3 Pharmacokinetic parameters in the Huh7 CDX mouse model.

Supplementary Table S4 Mab-A-Dxd preliminary toxicity study in cynomolgus monkey.

## Data Availability

Data generated in this study are available upon request to the corresponding author. Some supporting data are included in the Supplementary Materials.
