# Supplementary material for: Development of Glypican 3–Targeting Antibody–Drug Conjugates for Hepatocellular Carcinoma Therapy
Source: Cancer Res Commun. 2026 Jul 14;6(7):1665–80. doi: 10.1158/2767-9764.CRC-26-0139 (PMC13366411; doi:10.1158/2767-9764.CRC-26-0139)
Supplement: Supplementary Figure S1 — Bystander killing effects of Dxd-based ADCs. [file crc-26-0139_supplementary_figure_s1_suppsf1.docx]

**
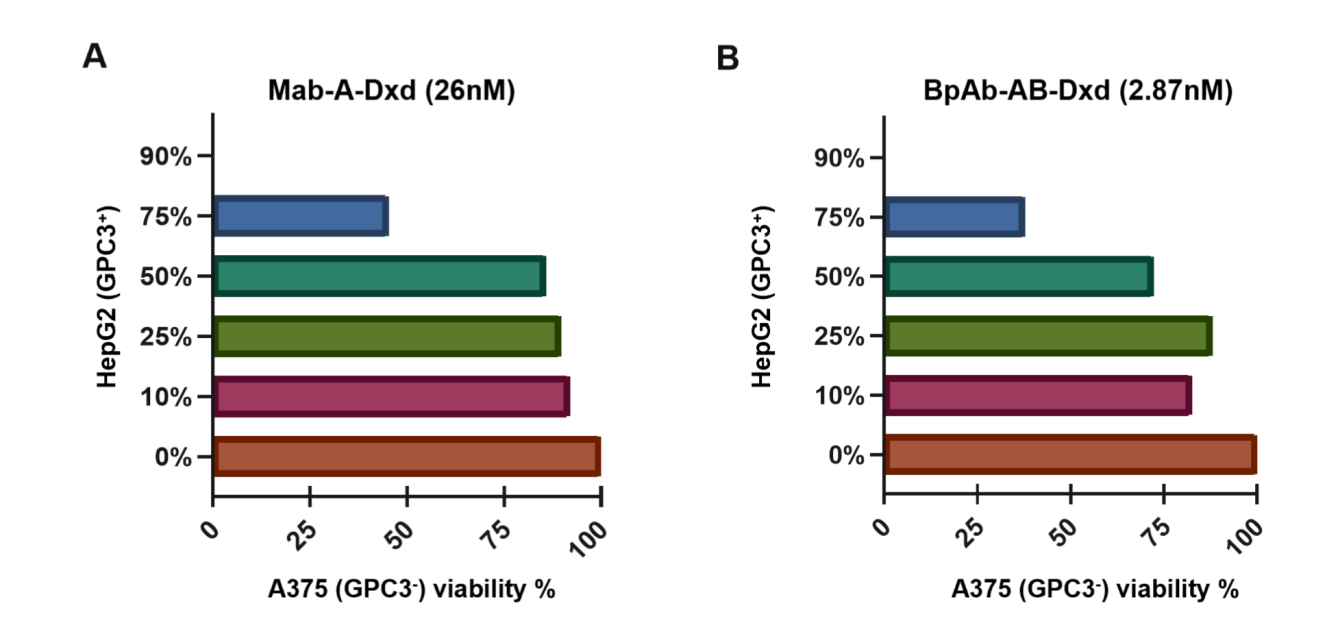
**

**Supplementary Figure S1. Bystander killing effects of Dxd-based ADCs.** (**A-B**) Bystander killing assay in which HepG2 (GPC3-positive) and A375 (GPC3-negative) cells were co-cultured at varying ratios. A375 cells were GFP-labeled, allowing cell viability to be quantified by GFP fluorescence. Both Mab-A-Dxd and BpAb-AB-Dxd exhibited clear bystander killing effects.
