# Supplementary material for: Development of Glypican 3–Targeting Antibody–Drug Conjugates for Hepatocellular Carcinoma Therapy
Source: Cancer Res Commun. 2026 Jul 14;6(7):1665–80. doi: 10.1158/2767-9764.CRC-26-0139 (PMC13366411; doi:10.1158/2767-9764.CRC-26-0139)
Supplement: Supplementary Figure S2 — Mab-A-Dxd exhibits superior pharmacokinetics and tumor suppression effect in the CDX model. [file crc-26-0139_supplementary_figure_s2_suppsf2.docx]

**
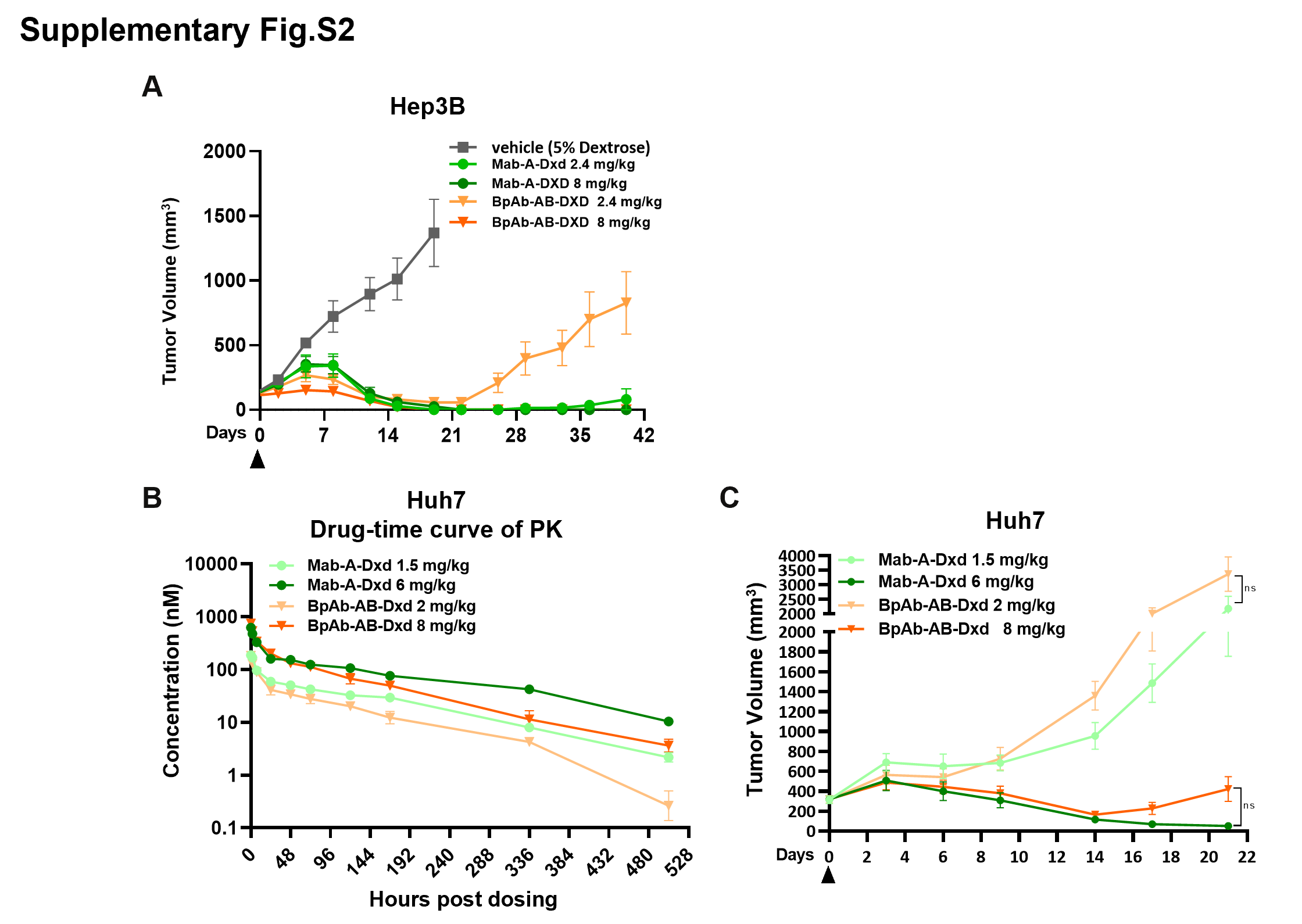
**

**Supplementary Figure S2. Mab-A-Dxd exhibits superior pharmacokinetics and tumor suppression effect in the CDX model.** **(A)** Extended observation of long-term antitumor efficacy in the Hep3B CDX model (based on Figure 4D) following treatment with Mab-A-Dxd and BpAb-AB-Dxd at 2.4 and 8 mg/kg. Data are shown as mean ± SD (n = 6). **(B-C)** In vivo blood concentration (total antibody) and corresponding antitumor activity of Mab-A-Dxd and BpAb-AB-Dxd in Huh7 tumor-bearing nude mice. Data are presented as mean ± SD (n = 3).
