# Supplementary material for: Development of Glypican 3–Targeting Antibody–Drug Conjugates for Hepatocellular Carcinoma Therapy
Source: Cancer Res Commun. 2026 Jul 14;6(7):1665–80. doi: 10.1158/2767-9764.CRC-26-0139 (PMC13366411; doi:10.1158/2767-9764.CRC-26-0139)
Supplement: Supplementary Figure S3 — Mab-A-Dxd displays antitumor activity in GPC3-positive HCC and NSCLC PDX models. [file crc-26-0139_supplementary_figure_s3_suppsf3.docx]

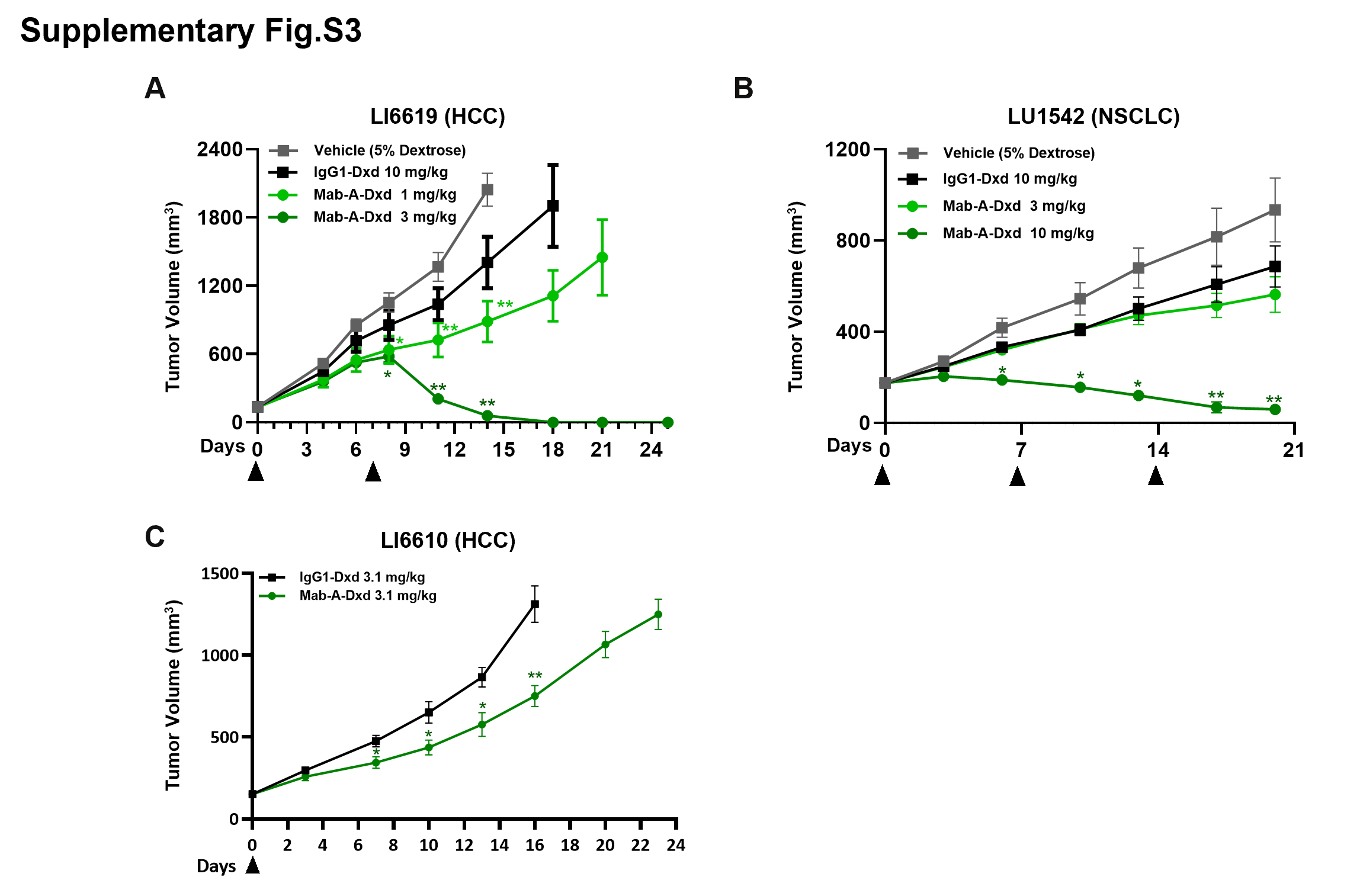


**Supplementary Figure S3. Mab-A-Dxd displays antitumor activity in GPC3-positive HCC and NSCLC PDX models.** **(A-B)** Same as Figure 5A and 5D, with the addition of an IgG1-Dxd isotype control. **(C)** Antitumor activity of Mab-A-Dxd at low dose (3.1 mg/kg) in the LI6610 HCC PDX model (low GPC3 H-score). Data are presented as mean ± SD (n = 5).
